# Structural and Kinetic Hydrogen Sorption Properties of Zr_0.8_Ti_0.2_Co Alloy Prepared by Ball Milling

**DOI:** 10.1155/2018/5736742

**Published:** 2018-03-12

**Authors:** Hui He, Huaqin Kou, Wenhua Luo, Tao Tang, Zhiyong Huang, Ge Sang, Guanghui Zhang, Jingwen Ba, Meng Liu

**Affiliations:** ^1^Science and Technology on Surface Physics and Chemistry Laboratory, P.O. Box 9072-35, Mianyang 621908, China; ^2^Institute of Materials, China Academy of Engineering Physics, P.O. Box 9071-12, Mianyang 621907, China; ^3^State Key Laboratory of Silicon Materials, Zhejiang University, Hangzhou 310027, China

## Abstract

The effects of ball milling on the hydrogen sorption kinetics and microstructure of Zr_0.8_Ti_0.2_Co have been systematically studied. Kinetic measurements show that the hydrogenation rate and amount of Zr_0.8_Ti_0.2_Co decrease with increasing the ball milling time. However, the dehydrogenation rate accelerates as the ball milling time increases. Meanwhile, the disproportionation of Zr_0.8_Ti_0.2_Co speeds up after ball milling and the disproportionation kinetics is clearly inclined to be linear with time at 500°C. It is found from X-ray powder diffraction (XRD) results that the lattice parameter of Zr_0.8_Ti_0.2_Co gradually decreases from 3.164 Å to 3.153 Å when the ball milling time extends from 0 h to 8 h, which is mainly responsible for the hydrogen absorption/desorption behaviors. In addition, scanning electron microscope (SEM) images demonstrate that the morphology of Zr_0.8_Ti_0.2_Co has obviously changed after ball milling, which is closely related to the hydrogen absorption kinetics. Besides, high-resolution transmission electron microscopy (HRTEM) images show that a large number of disordered microstructures including amorphous regions and defects exist after ball milling, which also play an important role in hydrogen sorption performances. This work will provide some insights into the principles of how to further improve the hydrogen sorption kinetics and disproportionation property of Zr_0.8_Ti_0.2_Co.

## 1. Introduction

Because of the rapid decrease of fossil fuels and the increasingly serious environmental pollution in recent years, developing a clean and renewable energy has become an urgent task for mankind [1]. Under the development of ITER (International Thermonuclear Experimental Reactor), fusion energy, by burning the fuel of deuterium (D) and tritium (T) plasma, is regarded as one of the most ideal energy sources due to its huge energy release, abundant fuel resources, and low radioactivity [2–4]. In order to ensure the successful operation of fusion reactors, a viable, highly efficient, safe, and inexpensive hydrogen isotope storage method for D-T fuel is very necessary [5, 6].

In ITER, the D-T fuel is recommended to be stored as metal deuteride and tritide because solid-state hydrogen isotopes storage offers such advantages as safety, efficiency (higher bulk hydrogen storage density), and processing convenience over gas and liquid storage methods [7, 8]. Among several alternative hydrogen storage materials, an intermetallic compound of ZrCo is proposed as one of the most suitable candidates for tritium storage according to (1), since it possesses such excellent properties as low equilibrium hydrogen pressure and fast hydrogen absorption rate at room temperature, moderate temperature for hydrogen desorption to 100 kPa, and desirable features of safety like nonradioactivation, low pyrophoricity, and small volume expansion during hydrogen sorption cycles [9–14].

(1) $$\begin{array}{c} 2{\text{ZrCoH}}_{\text{3}}⟷2\text{ZrCo}+3{\text{H}}_{2} \end{array}$$

However, its serious degradation of hydrogen storage properties during the hydrogen sorption cycle obstructs its wide application, resulting from the concomitant hydrogen-induced disproportionation reaction that happened above 573 K during hydrogen sorption process shown as follows[15–18]:

(2a) 2ZrCo+H2⟶ZrH2+ZrCo2

(2b) 2ZrCoH3⟶ZrH2+ZrCo2+2H2

Because of the high thermodynamic stabilities of ZrH_2_ and ZrCo_2_, the disproportionation of ZrCo will cause significant degradation of hydrogen storage properties during the practical hydrogen sorption cycles [19].

Recently, many investigations mainly focused on element substitution have been made to improve the antidisproportionation property of ZrCo, which achieved remarkable progress [20–24]. Compared with other elements, Ti has been surprisingly found to be the most effective substitution element for improving the antidisproportionation property so far. Huang et al. [25] found that the equilibrium hydrogen desorption pressure of Zr_1−*x*_Ti_*x*_Co alloy increases along with increasing Ti content. In the meantime, hydrogen sorption cycles do not produce separated ZrCo, TiCo, and ZrH_2_, suggesting that the antidisproportionation property of ZrCo alloy is improved by Ti substitution. Zhao et al. also confirmed that partial substitution of Ti for Zr will significantly enhance the antidisproportionation property of ZrCo [26, 27]. Zhang et al. [28] systematically compared the effects of Ti substitution on the disproportionation behaviors of ZrCo with Sc, Ni, and Fe elements. It was observed that Ti has superior effect on suppressing the disproportionation of ZrCo. Similar result was proved by Kou et al. who reported that Zr_0.8_Ti_0.2_Co bed had better durability against disproportionation than ZrCo bed and Zr_0.8_Hf_0.2_Co bed [29]. Meanwhile, Jat et al. [30] further investigated the influences of Ti amount on the disproportionation rate of Zr_1−*x*_Ti_*x*_Co alloy and found the disproportionation decreases in order of ZrCo > Zr_0.9_Ti_0.1_Co > Zr_0.7_Ti_0.3_Co > Zr_0.8_Ti_0.2_Co.

Although it seems that Ti-substituted ZrCo alloy possesses the improved antidisproportionation property, the requirements of practical application still cannot be completely satisfied due to its stagnant hydrogen sorption kinetics. Zhao et al. [26] reported that the hydrogen absorption amount and rate of ZrCo decrease with the Ti content increasing. Moreover, Shmayda et al. [31] and Yoo et al. [32] showed that the kinetic response of ZrCo for hydrogen sorption is slower than uranium, which restricts the application of ZrCo alloy. Therefore, it is of great necessity to further enhance the hydrogen sorption kinetics of Ti-substituted ZrCo alloy.

It has extensively shown that mechanical ball milling is an effective technique for improving the kinetic property of hydrogen storage materials [33–36]. However, the effects of ball milling on the hydrogen sorption kinetics and microstructure of ZrCo-based alloy have never been investigated. In this paper, we focused on the effects of ball milling on microstructure and kinetic hydrogen sorption properties of Zr_0.8_Ti_0.2_Co alloy. Furthermore, because study of kinetics is clearly beneficial to understanding of mechanism about hydrogen sorption process [20, 37, 38], kinetic analysis has also been carried out for disproportionation of Zr_0.8_Ti_0.2_Co in this work.

## 2. Experimental

### 2.1. Sample Preparation and Kinetic Measurements

Zr_0.8_Ti_0.2_Co powder with particle size of 2~4 mm was purchased from the General Research Institute for Nonferrous Metals (Beijing, China). The hydrogen sorption behaviors of the samples were investigated by a Sievert type apparatus. The detailed activation procedures of Zr_0.8_Ti_0.2_Co were performed according to [39]. The samples were prepared by ball milling of Zr_0.8_Ti_0.2_Co powder using a planetary mill at 400 rpm for different time. After ball milling, the samples were firstly evacuated at 500°C, and then the hydrogen absorption measurements of samples were conducted at 100°C under 0.8 bar H_2_. For each time, about 1 g sample was loaded in the reactor of the apparatus, and hydrogen pressure reduction of ~0.2 bar was achieved for hydrogenation. After hydrogenation, the hydrogen in the reactor was evacuated and then the dehydrogenation measurements of samples were carried out from room temperature to 500°C with heating rate of 5°C/min. After that, the isothermal disproportionation kinetics was examined at 500°C for more than 500 min. The change of hydrogen pressure (*P*) as a function of time was recorded from room temperature to the end of the isothermal period. During the whole dehydrogenation process, the initial hydrogen pressure was recorded as *P*_0_ and the maximum addition of hydrogen pressure was denoted as *P*_max_. As a result, the hydrogen desorption and disproportionation of the sample were quantified by formula as (*P* − *P*_0_)/*P*_max⁡_.

### 2.2. Structural Characterizations

X-ray powder diffraction (XRD) method was used to characterize the crystal structures of Zr_0.8_Ti_0.2_Co samples at different states on a DX2700B diffractometer with Cu K_a_ radiation, 40 kV, and 30 mA. The XRD patterns were recorded in steps of 0.02° (2*θ*) from 20° to 90° with a constant scanning rate of 0.6 s per step. For Rietveld refinement of lattice parameters, special XRD data was obtained by scanning rate of 1.8 s per step.

The morphology of the samples was studied by scanning electron microscope (SEM, Ultra55, CARL ZEISSNTS GmbH) and the compositional analysis on surface was carried out using energy dispersive X-ray spectroscopy (EDS, Inca). Morphological observation inside the particle was carried out by Helios Nanolab 600i Focused Ion Beam (FIB) using beam energy of 20 keV and a beam current of 2.8 nA. The microstructure of the samples was investigated using high-resolution transmission electron microscopy (HRTEM, Libra 200, CARL ZEISS IRTS) with an accelerating voltage of 200 kV.

## 3. Results and Discussion

### 3.1. Hydrogen Absorption/Desorption Kinetics

**Figure 1:**
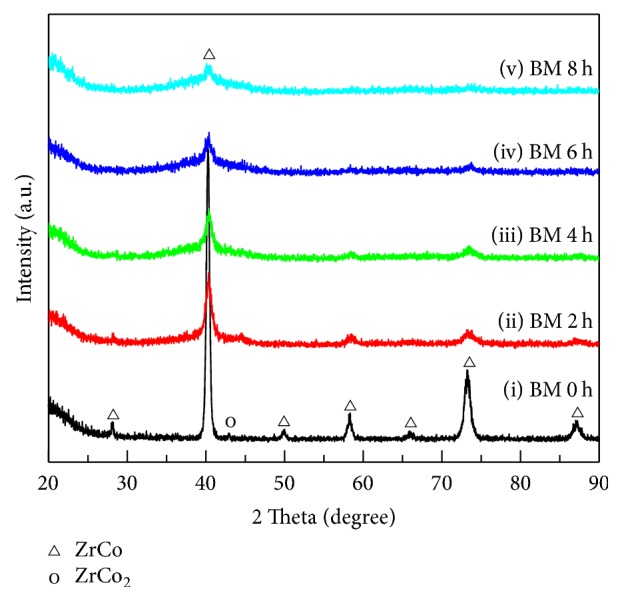
XRD patterns of the activated Zr_0.8_Ti_0.2_Co samples after ball milling for different time. (i) 0 h; (ii) 2 h; (iii) 4 h; (iv) 6 h; (v) 8 h.

Figure 1 represents the XRD patterns of activated Zr_0.8_Ti_0.2_Co samples after ball milling for different time. It can be found that Zr_0.8_Ti_0.2_Co samples still keep ZrCo phase except for trace amount of ZrCo_2_ phase, which is identical with previous researches [26, 27]. Meanwhile, it is clear that the intensities of ZrCo diffraction peaks decrease and the peaks exhibit broadened characteristics with increasing ball milling time. It is suggested that the crystalline structure of ZrCo phase may be damaged and the average grain size of samples should decrease during the ball milling process [40].

**Figure 2:**
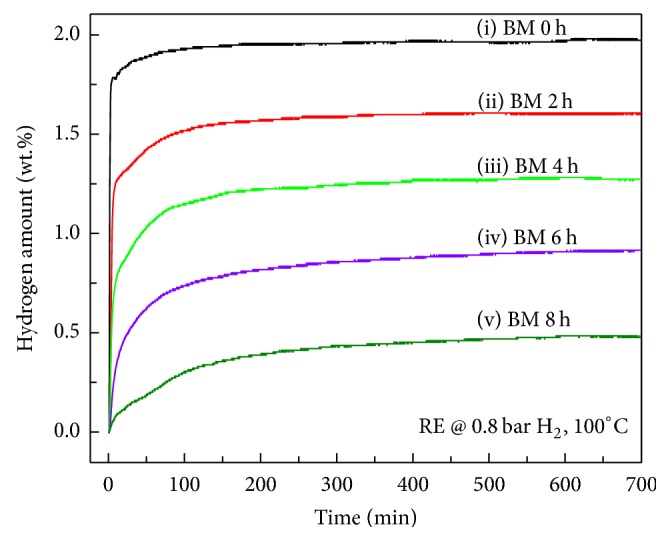
Hydrogen absorption curves of Zr_0.8_Ti_0.2_Co samples under 0.8 bar H_2_ at 100°C after ball milling for different time. (i) 0 h; (ii) 2 h; (iii) 4 h; (iv) 6 h; (v) 8 h.

The hydrogen absorption curves of Zr_0.8_Ti_0.2_Co samples after ball milling for different time are displayed in Figure 2. Since the disproportionation reactions of ZrCo alloy shown as (2a) and (2b) usually take place at high temperature, the curves in Figure 2 should represent the hydrogenation reaction shown as (1). Obviously, the hydrogen absorption amount decreases with increasing ball milling time. The hydrogenation amount reaches 1.97 wt.% for the sample without ball milling, whereas the total hydrogen amount of only 0.50 wt.% can be obtained for the sample ball milled for 8 h. Meanwhile, the hydrogen absorption rate also exhibits a tendency to slow down after ball milling.

**Figure 3:**
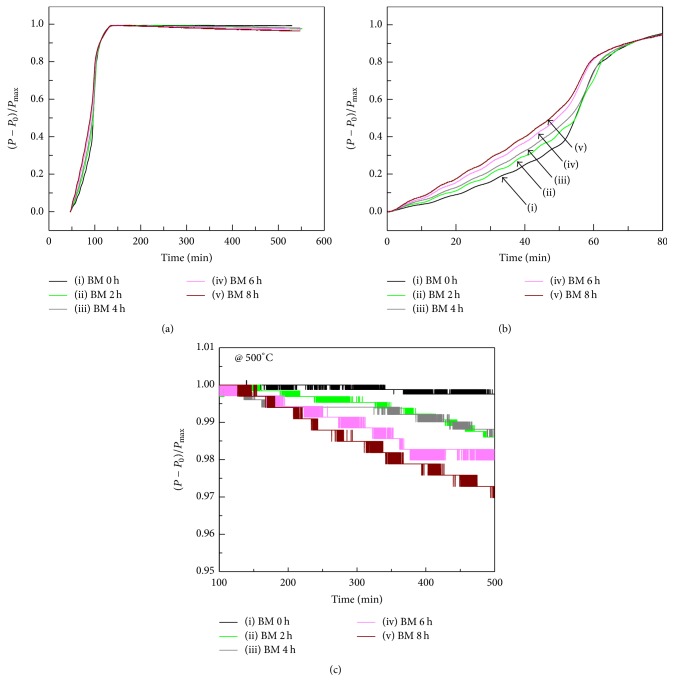
Dehydrogenation curves (a), enlarged dehydrogenation curves (b), and enlarged disproportionation curves (c) of Zr_0.8_Ti_0.2_Co samples ball milled for different time. (i) 0 h; (ii) 2 h; (iii) 4 h; (iv) 6 h; (v) 8 h.

The dehydrogenation curves of ball milled Zr_0.8_Ti_0.2_Co samples after hydrogenation are shown in Figure 3. According to (2a) and (2b), the disproportionation reaction displayed by (2b) only happens under high hydrogen pressure. However, the hydrogen pressure is lower than 1 bar in this study. Therefore, the disproportionation of Zr_0.8_Ti_0.2_Co sample only takes place according (2a) in this work, which causes the reduction in hydrogen pressure. As a result, the whole hydrogen desorption curve in Figure 3 can be divided into two stages, respectively, belonging to the temperature programmed dehydrogenation process (Figure 3(b)) and the disproportionation process (Figure 3(c)). It can be observed from the first stage that the hydrogen desorption rate is enhanced with increasing ball milling time, which is beneficial to practical application of ZrCo-based alloy. The second stage substantially represents an isothermal disproportionation process at 500°C. It can be seen that the disproportionation extent is very slight and the disproportionation rate is slow for all samples at 500°C, which is very close to the result reported by Zhang et al. [28]. Nevertheless, it is readily discernible that the disproportionation rate and extent monotonously grow with increasing ball milling time. These results suggest that not only the dehydrogenation rate but also the disproportionation rate of Zr_0.8_Ti_0.2_Co can be quickened by ball milling, the mechanism of which will be specifically discussed below.

**Figure 4:**
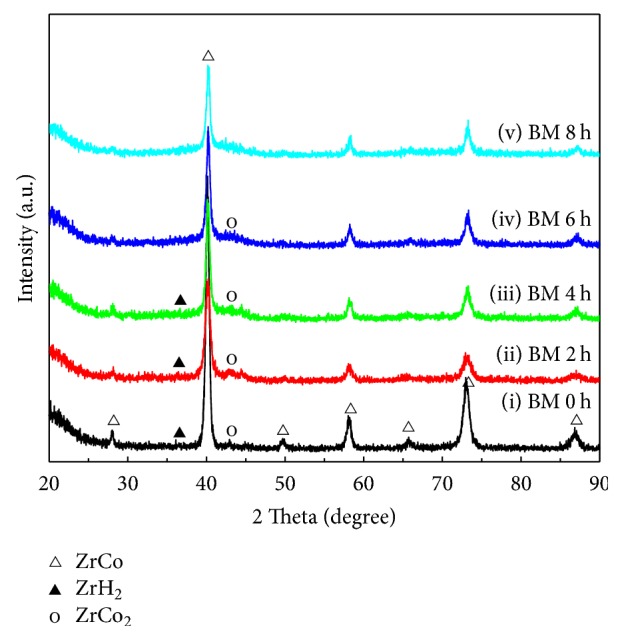
XRD patterns of the ball milled Zr_0.8_Ti_0.2_Co samples after disproportionation. (i) 0 h; (ii) 2 h; (iii) 4 h; (iv) 6 h; (v) 8 h.

Figure 4 demonstrates XRD patterns of the ball milled Zr_0.8_Ti_0.2_Co samples after disproportionation shown in Figure 3. It can be seen that the diffraction peaks corresponding to ZrCo phase are clearly present in all samples, though the intensity of ZrCo peaks decreases slightly with increasing ball milling time. The existence of major phase of ZrCo illustrates that the dehydrogenation mainly proceeds according to (1). In addition, the weak diffraction peaks of disproportionation products including ZrH_2_ and ZrCo_2_ can be carefully observed, indicating the disproportionation extent of Zr_0.8_Ti_0.2_Co sample should be small. The intensity of diffraction peaks for ZrH_2_ and ZrCo_2_ is well in agreement with the disproportionation behaviors displayed in Figure 3, which shows minor reduction in hydrogen pressure.

### 3.2. Structural Characterization and Kinetic Mechanism

**Figure 5:**
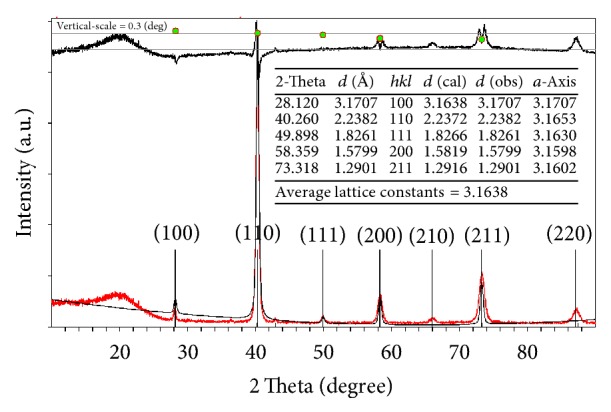
Rietveld refinement of X-ray diffraction pattern of Zr_0.8_Ti_0.2_Co sample without ball milling.

From the results above, it can be found that the hydrogen desorption kinetics and disproportionation kinetics of Zr_0.8_Ti_0.2_Co become faster, while the hydrogenation kinetics is restrained by ball milling. To comprehend these kinetic behaviors, detailed structural characterizations were performed. Firstly, the phase structure variation after ball milling has been investigated. To determine the lattice parameters of Zr_0.8_Ti_0.2_Co samples as accurately as possible, special XRD experiments have been performed to obtain strong diffraction signal by increasing the scanning time. According to the XRD results, the lattice parameters of the samples have been calculated by Rietveld refinement of XRD data via Jade 6.0 software. The representative Rietveld refinement pattern of Zr_0.8_Ti_0.2_Co sample is shown in Figure 5. The refinement result shows that the crystal structure of Zr_0.8_Ti_0.2_Co sample without ball milling is CsCl-type cubic (bcc) with lattice parameter *a* = 3.1638 Å, which is in good agreement with other study [28]. According to the specific Rietveld refinement results as displayed in Table 1, the lattice parameter and cell volume of Zr_0.8_Ti_0.2_Co sample slightly and continuously decrease with increasing ball milling time, which may be probably attributed to cumulative plastic deformation and microstrain in the crystal lattice during the ball milling process [33, 40]. In view of the variation of lattice parameter, it is easy to understand the kinetic performances of Zr_0.8_Ti_0.2_Co after ball milling. When the lattice parameter and cell volume become smaller, the occupancy of H atom in interstitial sites will become more difficult but release of H atom from interstitial sites will be easier [41]. Hence, it is observed that hydrogen absorption kinetics decreases but hydrogen desorption kinetics increases for Zr_0.8_Ti_0.2_Co with increasing ball milling time.

**Figure 6:**
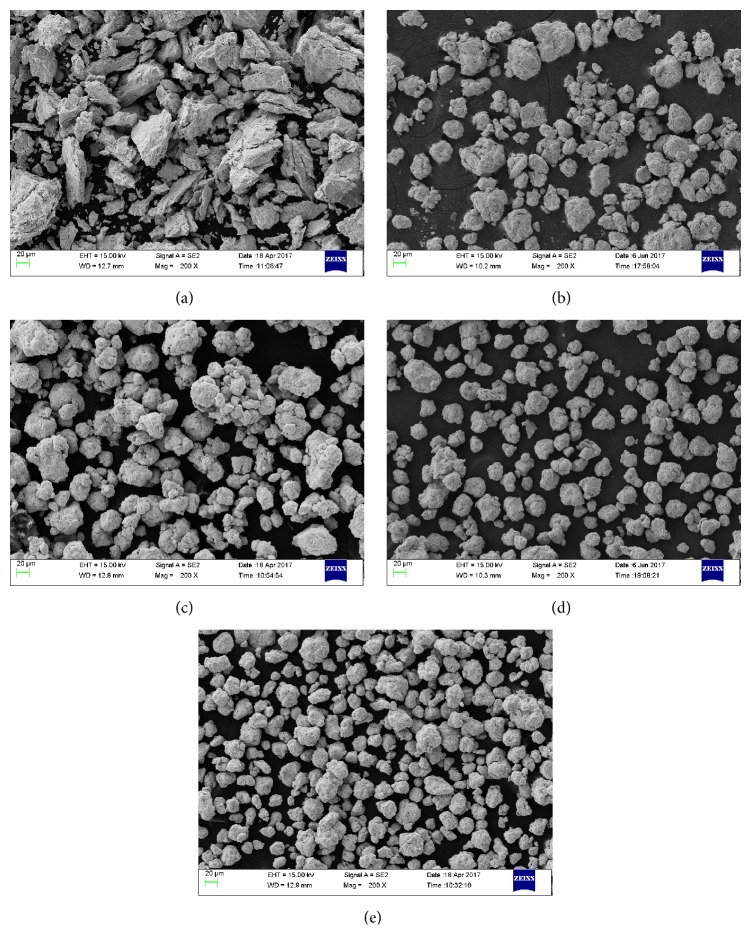
SEM images of the Zr_0.8_Ti_0.2_Co samples ball milled for different time. (a) 0 h; (b) 2 h; (c) 4 h; (d) 6 h; (e) 8 h.

To investigate the morphologies of ball milled Zr_0.8_Ti_0.2_Co samples, SEM analysis has been performed and the results are shown in Figure 6. After ball milling, the morphologies of Zr_0.8_Ti_0.2_Co sample tend to be small particles from initial chip. Meanwhile, the particle size gradually decreases with increasing the ball milling time. Besides, as the ball milling time increases, the agglomeration of particles is distinctly reduced and the particles become more dispersive.

**Figure 7:**
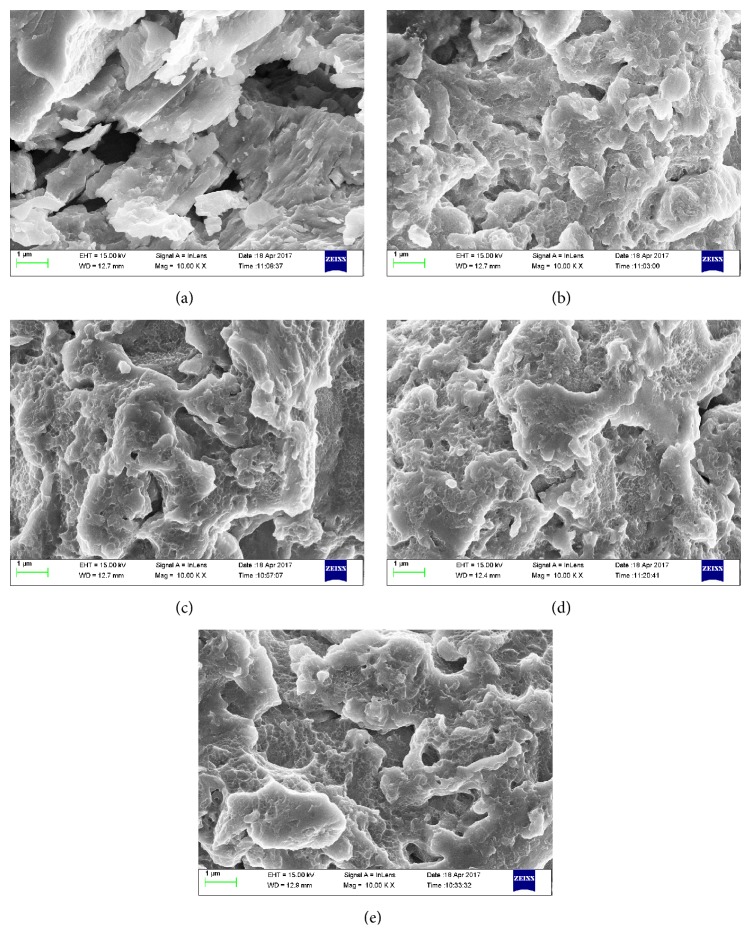
SEM images of the Zr_0.8_Ti_0.2_Co samples after ball milling for different time at magnification of 10000x. (a) 0 h; (b) 2 h; (c) 4 h; (d) 6 h; (e) 8 h.

Furthermore, in order to closely observe the surface of the samples, the SEM images of the ball milled Zr_0.8_Ti_0.2_Co samples at magnification of 10000x were obtained, as shown in Figure 7. A lot of chips and sharp edges are observed for the sample without ball milling. However, the sharp edges can hardly be observed on the surface of ball milled Zr_0.8_Ti_0.2_Co sample, which should be caused by deformation and compaction during the ball milling process. It is well known that the edges are apt to break off and fresh surface will emerge in the process of hydrogenation [42, 43], which is beneficial to enhancement of hydrogen absorption. Moreover, as a direct and fast diffusion path of hydrogen, the edges are also beneficial to fast hydrogen absorption [44]. Hence, the change in morphology is mainly responsible for the decrease of hydrogen absorption kinetics for Zr_0.8_Ti_0.2_Co samples after ball milling.

**Figure 8:**
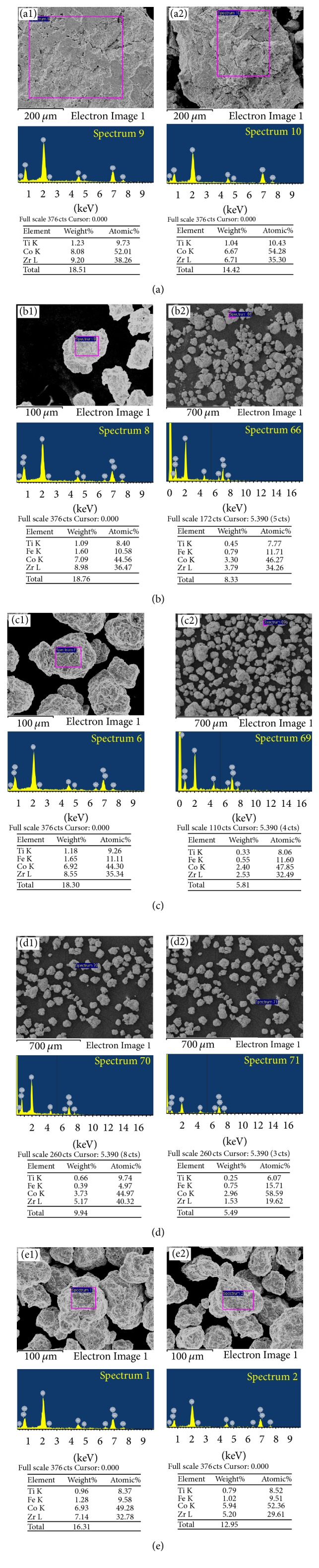
EDS results of the Zr_0.8_Ti_0.2_Co samples after ball milling for different time. (a) 0 h; (b) 2 h; (c) 4 h; (d) 6 h; (e) 8 h.

In order to analyze the composition of the sample surface, two representative EDS spectrums of every sample over the surface were obtained, as shown in Figure 8. The detailed elemental contents for every sample are listed in Table 2. It is interesting to discover that the existence of Fe element on the surface of particles is detected for all ball milled Zr_0.8_Ti_0.2_Co samples. This suggests that the Fe element may be brought by steel milling vessel and balls during the ball milling process [38]. It has been reported that the disproportionation of ZrCo will be accelerated after doping Fe [22, 28]. Hence, it is reasonable to believe that faster disproportionation rate of ball milled Zr_0.8_Ti_0.2_Co samples has direct correlation with the introduced Fe element on the surface of particles. Besides, it can be found that the average atom ratio of (Zr + Ti)/(Co + Fe) for the selected regions decreases gradually from 0.89 to 0.66 when the ball milling time extends from 0 h to 8 h. As previous studies proved, when the average atom ratio of Zr : Co is much closer to 0.5 (1 : 2), the elemental recombination of ZrCo_2_ phase will be facilitated during the disproportionation process [45, 46]. Consequently, disproportionation of ZrCo is aggravated. Due to the analogical feature of group element, the substitution element of Ti and introduced Fe can, respectively, be included in Zr and Co. As a result, the gradual decrease of (Zr + Ti)/(Co + Fe) from 0.89 to 0.66 may be another reason that aggravates the disproportionation of Zr_0.8_Ti_0.2_Co sample by increasing ball milling time, as shown in Figure 3.

**Figure 9:**
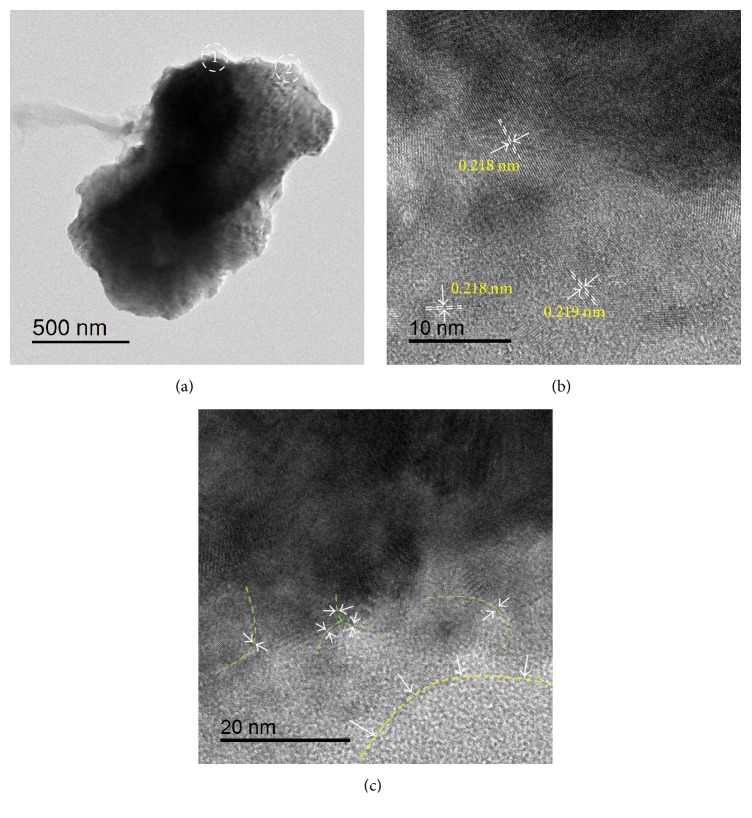
TEM analysis of Zr_0.8_Ti_0.2_Co sample for ball milled for 8 h. (a) Low-magnification image of particle; (b) high-resolution image of peripheral zone 1 in (a); (c) high-resolution image of peripheral zone 2 in (a).

In order to further characterize the microstructure, the TEM analysis for Zr_0.8_Ti_0.2_Co samples ball milled for 8 h has been employed and the results are shown in Figure 9. Figures 9(b) and 9(c), respectively, show enlarged TEM images and corresponding SAED patterns of zones 1 and 2 in Figure 9(a). It can be found from the SAED patterns that the Zr_0.8_Ti_0.2_Co particle ball milled for 8 h is comprised of not only polycrystalline regions but also a number of amorphous regions. In accordance with PDF number 030657272, the interplanar distances of 0.218 nm, 0.218 nm, and 0.219 nm in Figure 9(b) all correspond to {110} planes of Zr_0.8_Ti_0.2_Co. According to Figure 5, the interplanar distance of {110} should be 0.224 nm for Zr_0.8_Ti_0.2_Co sample without ball milling. It is clear that the interplanar distance of {110} has been shortened to be about 0.218 nm after ball milling for 8 h. It is suggested that the lattice parameter of Zr_0.8_Ti_0.2_Co sample was decreased after ball milling, which is in good agreement with the Rietveld refinement results of XRD. Moreover, a number of amorphous regions (over yellow line) are clearly observed in Figure 9(c). Meanwhile, a large number of defects including dislocations and grain boundaries (around green line) are widely observed, which provide a possible explanation for the broadened XRD patterns shown in Figure 1. As well known, these disordered microstructures mentioned above are favorable for H atom diffusion instead of occupation [38, 47]. Therefore, the reduction of the hydrogenation amount and enhancement of the dehydrogenation rate for ball milled Zr_0.8_Ti_0.2_Co samples are probably resulting from the disordered microstructures produced during the ball milling process.

### 3.3. Kinetic Model Analysis

**Figure 10:**
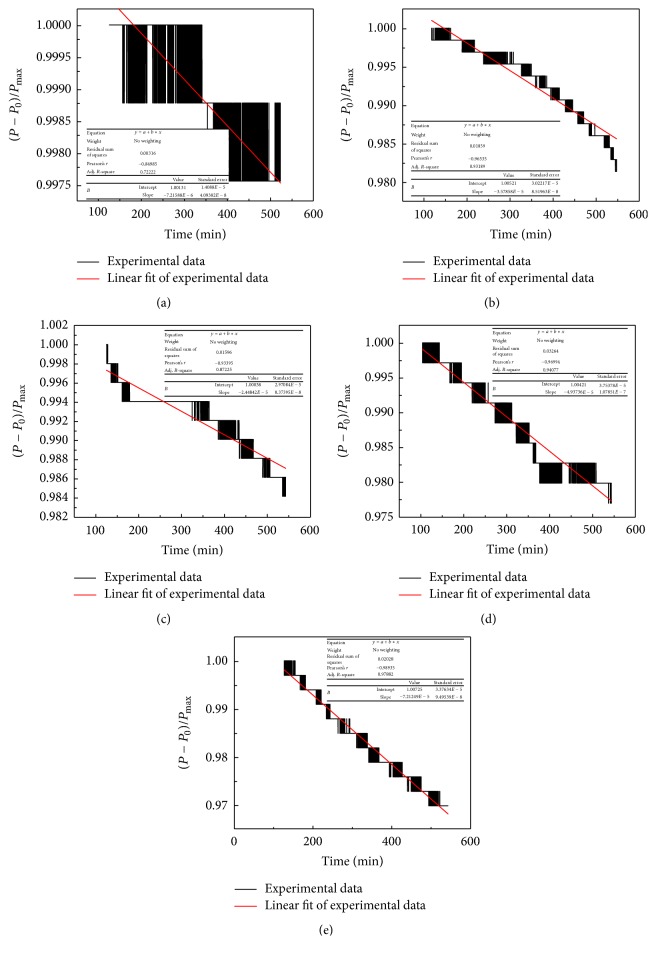
The linear fitting results of disproportionation kinetics for Zr_0.8_Ti_0.2_Co samples after ball milling for different time. (a) 0 h; (b) 2 h; (c) 4 h; (d) 6 h; (e) 8 h.

In order to further understand the disproportionation kinetic mechanism, the kinetic analysis of disproportionation has been performed for ball milled Zr_0.8_Ti_0.2_Co. Usually, the relationship between the reacted fraction and reaction time is linear for surface reaction controlled step [48, 49]. According to Figure 3, it can been found that the disproportionation curves of all Zr_0.8_Ti_0.2_Co samples should just represent the initial stage of whole disproportionation and the reaction equilibrium has not been reached within 450 min at 500°C. Moreover, the disproportionation kinetics of all Zr_0.8_Ti_0.2_Co samples in this stage is clearly inclined to be linear. Thus, the isothermal disproportionation data of Zr_0.8_Ti_0.2_Co sample after ball milling for different time has been linearly fitted, as shown in Figure 10. It can be seen from the fitting results that the disproportionation kinetics of Zr_0.8_Ti_0.2_Co samples is much closer to linear with increasing the ball milling time. Meanwhile, the slope of fitted line is slightly decreased, corresponding to the slight aggravation of disproportionation rate. The well-fitted linear results suggest that the disproportionation stage of Zr_0.8_Ti_0.2_Co samples curved by Figure 3 should be a surface reaction controlled step.

Based on the results of microstructure and kinetic analysis above, some useful insights can be obtained into the mechanism of hydrogen sorption reaction and disproportionation reaction for ball milled Zr_0.8_Ti_0.2_Co samples. The effects of ball milling on the hydrogen sorption and disproportionation behaviors of Zr_0.8_Ti_0.2_Co sample can be deduced from the changed microstructure including lattice parameter, morphology, elemental composition, and crystal defects. For hydrogen sorption reaction, it can be found that ball milling shows negative effect on the hydrogen absorption rate of Zr_0.8_Ti_0.2_Co sample, which is different from the commonly positive effects of ball milling on hydrogen storage materials in many studies. The negative effects of ball milling may be resulting from three aspects: (1) the decreased cell volume makes the entering of hydrogen atom into interstitial site in lattice more difficult; (2) the missing sharp edges of particles cut the paths for fast transfer of hydrogen; (3) the increased disorderliness of microstructure goes against the occupation of hydrogen. As a result, it was observed that the hydrogen absorption rate and amount of Zr_0.8_Ti_0.2_Co sample were decreased by increasing ball milling. On the other hand, it can be found that the ball milling plays a positive role in hydrogen desorption rate. The positive effects of ball milling may be derived from three points: (1) the decreased cell volume results in the fact that H atoms begin to be less-stable in the crystal lattice and are inclined to leave from crystal interstitial sites; (2) the introduced Fe on the surface may act as catalysts for hydrogen dissociation [50]; (3) the produced lattice defects like dislocations and grain boundaries possibly act as a pathway for hydrogen transportation during dehydrogenation [51, 52]. Consequently, it was seen that the hydrogen desorption rate was increased by ball milling.

For disproportionation reaction, it is found the disproportionation rate of Zr_0.8_Ti_0.2_Co sample was exacerbated by ball milling. According to the kinetic analysis, the disproportionation stage of Zr_0.8_Ti_0.2_Co in this work should be a surface reaction controlled step, indicating that the surface state is very important for the disproportionation. As mentioned in the analysis of EDS results, Fe element was introduced during the ball milling process on the surface of Zr_0.8_Ti_0.2_Co particles, resulting in the gradual decrease of (Zr + Ti)/(Co + Fe). This possibly facilitates the elemental recombination of ZrCo_2_ phase. So, it is observed that the ball milled Zr_0.8_Ti_0.2_Co owns faster disproportionation kinetics. On the other hand, it is found from the microstructure that considerable crystal defects and strain energy were formed after ball milling, especially on the surface. It is well known that the crystal defects and strain energy will facilitate the transfer process of hydrogen atoms during hydrogen sorption reaction, which may be also beneficial for promoting the transfer of hydrogen atom from ZrCo to form ZrH_2_. Consequently, it is observed that the rate of the initial disproportionation stage for Zr_0.8_Ti_0.2_Co is enhanced by ball milling. In conclusion, if Zr_0.8_Ti_0.2_Co is expected to not only own fast hydrogen sorption kinetics but also have good antidisproportionation, it may be a good way to prepare Zr_0.8_Ti_0.2_Co as fine powder with smaller lattice parameter, uniform element distribution, and orderly microstructure and without impurity.

## 4. Conclusion

In summary, the effects of ball milling on the hydrogen sorption properties and microstructure of Zr_0.8_Ti_0.2_Co have been investigated systematically. Experimental results show that hydrogen absorption kinetics of Zr_0.8_Ti_0.2_Co decreases and the hydrogen desorption kinetics accelerates with increasing the ball milling time. Meanwhile, the disproportionation rate of Zr_0.8_Ti_0.2_Co is aggravated after ball milling. Characterizations of microstructure reveal that the variation of hydrogen sorption kinetics for Zr_0.8_Ti_0.2_Co after ball milling is mainly resulting from the changed microstructure including lattice parameter, morphology, elemental composition, and crystal defects. Kinetic analysis reveals that the initial stage of disproportionation for Zr_0.8_Ti_0.2_Co is a surface step controlled reaction. And the slight aggravation of disproportionation rate may be attributed to the introduced Fe on the surface of particles and the crystal defects together with strain energy after ball milling.

## Figures and Tables

**Table 1:** Rietveld refinement results of XRD patterns of Zr_0.8_Ti_0.2_Co samples after ball milling for different time.

System	Ball milling time/hour	Space group	Lattice parameters of major constituent phase (ZrCo phase)/Å	Cell volume of major constituent phase (ZrCo phase)/Å^3^
ZrCo [13]	0	*Pm-3m*	3.1957	32.64
Zr_0.8_Ti_0.2_Co	0	*Pm-3m*	3.1638	31.67
	2		3.1587	31.55
	4		3.1574	31.48
	6		3.1555	31.42
	8		3.1534	31.35

**Table 2:** EDS results of elemental composition for selected regions of Zr_0.8_Ti_0.2_Co samples after ball milling for different time.

ZrCo samples	Selected region	Zr (atomic%)	Ti (atomic%)	Co (atomic%)	Fe (atomic%)	(Zr + Ti)/(Co + Fe)
BM 0 h	1#	38.26	9.73	52.01		
	2#	38.49	7.48	54.04		
	Average	38.38	8.61	53.03		0.89

BM 2 h	1#	36.47	8.40	44.56	10.58	
	2#	34.26	7.77	46.27	11.71	
	Average	35.37	8.09	45.42	11.15	0.77

BM 4 h	1#	35.34	9.26	44.3	11.11	
	2#	32.49	8.06	47.85	11.60	
	Average	33.92	8.66	46.08	11.36	0.74

BM 6 h	1#	37.43	8.71	45.97	7.90	
	2#	28.83	7.2	56.89	7.08	
	Average	33.13	7.96	51.43	7.49	0.70

BM 8 h	1#	32.78	8.37	49.28	9.58	
	2#	29.61	8.52	52.36	9.51	
	Average	31.20	8.45	50.82	9.55	0.66
